# Increasing Soluble P-Selectin Levels Predict Higher Peripheral Atherosclerotic Plaque Progression

**DOI:** 10.3390/jcm12206430

**Published:** 2023-10-10

**Authors:** Philip Sommer, Michael Schreinlechner, Maria Noflatscher, Daniela Lener, Fabian Mair, Markus Theurl, Rudolf Kirchmair, Peter Marschang

**Affiliations:** 1 Department of Internal Medicine III (Cardiology, Angiology), Medical University of Innsbruck, Anichstr. 35, A-6020 Innsbruck, Austria; philip.sommer@tum.de (P.S.);; 2 Department of Internal Medicine, Central Hospital of Bolzano (SABES-ASDAA), Via Lorenz Boehler 5, I-39100 Bolzano, Italy

**Keywords:** atherosclerosis, 3D ultrasonography, P-selectin, plaque progression

## Abstract

Background and aims: The adhesion molecule P-selectin is expressed by endothelial cells and platelets. It is involved in platelet activation and leukocyte adhesion, both important processes in the pathogenesis of atherosclerosis. Our study was designed to assess the predictive value of soluble P-selectin (sP-selectin) on the progression of peripheral atherosclerosis. Methods: This is an observational, single-center, cohort study that included 443 patients with established cardiovascular disease (CVD) or at least one cardiovascular risk factor. Over a period of 4 years, each patient underwent three-dimensional (3D) ultrasound to assess the plaque volume of the carotid and femoral arteries once per year. In addition, plasma sP-selectin levels were measured at each visit. The association between changes in sP-selectin and peripheral atherosclerotic plaque progression was assessed using growth curve models. Results: 338 patients were available for statistical analysis. Each standard deviation increase in sP-selectin was significantly (*p* < 0.001) associated with a 46.09 mm^3^ higher plaque volume. In ROC-analysis, changes in sP-selectin over time showed an optimal cut-off value around Δ 0.0 µg/mL sP-selectin and significantly improved the predictive value of the ESC-SCORE (AUC for the combination of both parameters was 0.75 (95% CI 0.68–0.81, *p* < 0.001). Patients with increasing sP-selectin showed a significantly higher plaque progression compared to patients with decreasing or stable sP-selectin levels (202 mm^3^ vs. 110 mm^3^, *p* < 0.001). Conclusions: Increasing sP-selectin levels can predict higher atherosclerotic plaque progression as measured by 3D ultrasound. We suggest serial measurements of sP-selectin as an easily measurable biomarker for peripheral atherosclerotic plaque progression.

## 1. Introduction

In developed nations, cardiovascular diseases continue to be the leading cause of death [1]. The vast majority of cardiovascular events, such as myocardial infarction, stroke, or heart failure, occur on the basis of atherosclerosis. Atherosclerosis is a chronic disease driven by multiple pathophysiologic factors such as inflammation, lipid accumulation, oxidative stress, insulin resistance, and others [2]. In particular, inflammation is involved in virtually every step of pathogenesis [3]. Oxidized low-density lipoprotein (LDL) is believed to damage healthy endothelial cells and induce the expression of adhesion molecules on their surface [4]. These molecules induce the adhesion of leukocytes to the intima layer of the blood vessels [5]. One important group of adhesion molecules on the surface of intima cells are the endothelial cell-expressed selectins (E-selectin, P-selectin). These molecules mediate leukocyte rolling along the vessel wall until intercellular adhesion, molecule-1, and vascular cell adhesion molecule-1, induce firm adhesion followed by the transmigration of leukocytes across the endothelial layer [6].

Over the past few decades, many inflammatory biomarkers have been proposed as potential indicators of the progression of atherosclerotic plaques, aligning with the concept of atherosclerosis as an inflammatory disease [7,8,9]. High-sensitivity C-reactive protein (hsCRP) is the most extensively investigated inflammatory biomarker, and its use was recommended in the 2016 guidelines of the European Society of Cardiology (ESC) [10]. In the 2021 ESC guidelines on cardiovascular disease prevention, however, it is stated that hsCRP appears to have limited additional value in terms of reclassification potential [11]. Nevertheless, the ESC highlights cardiac biomarkers as a promising approach and recommends further research on this subject [11].

Several studies suggest that soluble P-selectin (sP-selectin) may be an even better biomarker than hsCRP since it is produced by platelets and endothelial cells, two cell types primarily involved in the pathogenesis of atherosclerosis [6]. Besides its role in the pathogenesis of atherosclerosis, P-selectin has also been proposed as a biomarker for arterial as well as venous thrombosis [12]. In particular, sP-selectin has been identified as a biomarker for cancer-associated thrombosis [13]. In patients with COVID-19, sP-selectin levels are elevated, and it is suggested that the upregulation of sP-selectin in COVID-19 patients might contribute to COVID-19 coagulopathy [14]. In a previously published study on CAD patients, we showed that the statin-induced reduction in sP-selectin correlated inversely with the progression of CAD [6]. In the literature, a large body of evidence highlights the role of P-selectin in the pathogenesis of atherosclerosis [15,16,17,18,19]. A secreted form of the molecule, sP-selectin, is circulating in plasma and has been measured in different pathophysiological conditions [20]. In a study by Carnevale et al., sP-selectin levels were higher in patients after myocardial infarction than in patients with stable angina pectoris [20]. Likewise, coronary thrombi showed higher P-selectin levels than the intracoronary blood of patients with stable angina pectoris [20].

For the evaluation of sP-selectin and its predictive value, we used a new ultrasound technology as a marker of subclinical atherosclerosis. Ultrasound has several advantages compared to other imaging techniques, including broad availability, relatively low cost, and a lack of radiation or contrast medium. The best studied sonographic parameter of subclinical atherosclerosis is the intima media thickness (IMT), which is considered to be a surrogate parameter for future cardiovascular events [21,22,23]. In a meta-analysis including 119 studies and over 100,000 patients, it was shown that lower progression of carotid intima-media thickness (cIMT) is associated with lower cardiovascular risk [24]. Compared to IMT, atherosclerotic plaques may be an even better predictor for future cardiovascular events [25]. Three-dimensional (3D) ultrasound to determine plaque volume was introduced by Sillesen and colleagues in the high-risk plaque BioImage study. In this study, performed on more than 6000 asymptomatic patients, carotid plaque burden was found to correlate stronger with coronary calcium score than other non-invasive parameters like the IMT [26]. Recently, automated software capable of precisely quantifying plaques has been developed, which makes the exact quantification of plaque volume within a relatively short time possible. Previous publications show that 3D plaque volumetry is a useful tool for the evaluation of cardiovascular biomarkers [27,28]. Here we report the association between sP-selectin and peripheral atherosclerotic plaque progression, which had been designed as a pre-specified endpoint of our study. Moreover, we investigated whether sP-selectin may be an efficient predictor for subclinical atherosclerotic plaque progression and whether it might significantly improve the predictive value of current risk scores, e.g., the SCORE of the ESC.

## 2. Materials and Methods

### 2.1. Study Design

**Figure 1:**
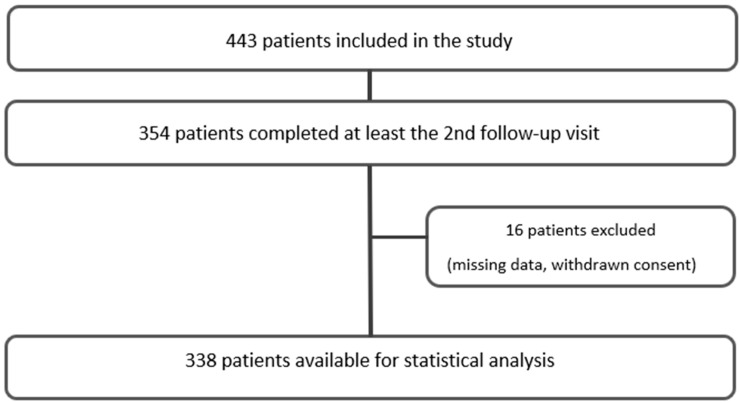
Flow chart of the study participants.

The study “Correlation of atherosclerotic Plaque Volume and Intima Media Thickness with soluble P-selectin” (ClinicalTrials.gov identifier: NCT01895725) is a prospective observational single-center cohort study designed and powered to evaluate the effect of sP-selectin on subclinical atherosclerosis. The study population’s baseline characteristics and the criteria for inclusion/exclusion have been published previously [29]. Briefly, individuals of both genders aged between 30 and 85 who had at least one traditional cardiovascular risk factor (CVRF) or a history of established cardiovascular diseases such as coronary artery disease (CAD), cerebrovascular disease (CBVD), or peripheral artery disease (PAD) were eligible for inclusion. Traditional CVRF was defined as arterial hypertension, dyslipidemia, current smoking status, positive family history, and pre-existing diabetes. The patient screening occurred at the outpatient clinic of the Department of Internal Medicine III (cardiology and angiology) at the Medical University of Innsbruck from 2013 to 2018. At baseline, each patient was subjected to ultrasound measurements, a physical examination, and routine laboratory tests. At the same time, the medical background on cardiovascular diseases, risk factors, concurrent medical conditions, ongoing medication, and the individual’s smoking status were recorded. Follow-up visits with re-examinations were carried out once per year over a period of four years. A total of 443 patients were included in this study. Of these, 354 patients completed at least the 2nd follow-up (354 patients 2nd follow-up, 280 patients 3rd follow-up, 190 patients 4th follow-up). Due to missing data and withdrawn consent, 338 patients were available for statistical analysis (Figure 1).

### 2.2. Ultrasound Imaging, Routine Laboratory, and Additional Examination

Ultrasound imaging included measurements of the intima-media-thickness and the plaque volume in the carotid arteries as well as in the femoral arteries on both sides. The IMT measurements were conducted using a Philip iU22 system, which was equipped with a linear L9-3 probe and integrated software for automatically calculating the mean IMT. In accordance with the Mannheim consensus, IMT measurements were taken at a location situated at least 1 cm proximal to the flow divider, within a plaque-free segment spanning 10 mm [30]. Plaques were defined as local formations that extended a minimum of 0.5 mm into the arterial lumen, occupied 50% of the adjacent IMT, or exhibited a thickness exceeding 1.5 mm from the media-adventitia boundary to the intima-lumen interface [30]. Plaque volume measurement was conducted using the same ultrasound system, which was equipped with a VL13-5 3D probe and specialized software for quantifying plaque volume in the bifurcation area and the visible sections of the internal and common carotid arteries. Identical measurements were performed in the femoral bifurcation and the adjacent sections of the common and superficial femoral arteries. The method’s reliability was assessed by computing the inter-observer variability among three different observers. The results revealed very good agreement among the raters, as indicated by an intra-class correlation coefficient of 0.95 (95% CI, 0.82–0.99).

The routine laboratory included measurements of total cholesterol, triglycerides, low-density lipoprotein (LDL) cholesterol, high-density lipoprotein (HDL) cholesterol, hs-CRP, fasting glucose, HbA1C, and creatinine levels. The estimated glomerular filtration rate (eGFR) was calculated using the Chronic Kidney Disease Epidemiology Collaboration (CKD-EPI) formula. Furthermore, an additional EDTA whole blood sample underwent centrifugation to separate the cellular components from the EDTA plasma. In the obtained EDTA plasma, sP-selectin levels were measured by means of a commercially available ELISA (Human P-Selectin/CD62P Quantikine ELISA Kit, R&D Systems, Minneapolis, MN, USA).

At baseline and during each follow-up examination, measurements of blood pressure, ankle brachial index (ABI), and pulse wave velocity (PWV) were performed. To measure ABI and pulse wave velocity, we used an automated system (AngE Pro4, SOT Medical Systems, Maria Rain, Austria). Furthermore, the ESC-SCORE (low risk chart) was calculated for each patient, and the patients were assigned to the corresponding risk categories (low/intermediate/high) following the recommendations of the ESC [10,31].

### 2.3. Statistical Analysis

The normal distribution was assessed using the Kolmogorov–Smirnov test. Continuous variables are presented as either mean ± standard deviation (SD) for parameters that exhibit a normal distribution or as median with the corresponding interquartile range (IQR) for parameters that do not conform to a normal distribution. Categorical variables are presented as absolute numbers and percentages. Total plaque volume (TPV) was defined as the combined plaque volume in the femoral and carotid arteries on both sides. For the sample size calculation, the linear correlation between biomarkers and atherosclerotic plaque progression was estimated. In a previous study, a reduction in soluble P-selectin was found to negatively correlate with the progression of coronary artery disease as measured by electron beam computed tomography (r^2^ = 0.393). Assuming comparable progression in the present study using ultrasound measurements, a possible correlation between P-selectin and atherosclerosis progression was calculated between r^2^ = 0.25 and 0.5. Therefore, for the lower margin of r (r = 0.25) with an alpha error of 0.05 and a power of 0.80, a minimum of 250 subjects would have been required. To account for possible problems with image quality, missing data, and patient dropout, a total of 600 subjects were originally planned for inclusion in this study. To assess differences in baseline parameters, we employed either a two-tailed, independent sample *t*-test or the Mann–Whitney U test for continuous variables and the χ^2^ test for categorical variables. To determine the effect of the number of cardiovascular diseases (none, 1, 2, 3) on the P-selectin levels, a mixed ANOVA with repeated measures was performed using the Greenhouse–Geisser correction. To examine the association between P-selectin and TPV over time, growth curve modeling was performed. The multilevel analysis contained two levels, with repeated observations of TPV on level 1 and individual subjects on level 2. After establishing the growth curve model, sP-selectin was added as a covariate. The model was expanded by stagewise adding demographics, CVRFs, and laboratory parameters as additional covariates. For the analysis of covariate variance, the maximum-likelihood estimation was used. Since at least two time points of plaque progression are necessary for the calculation of the regression curves, only patients who completed at least the 2nd follow-up visit were included in our analysis. Similar calculations were performed for hsCRP. For further statistical examination, the study cohort was split into a high total plaque progression (High-TPP) group and a low plaque progression group (Low-TPP), with the division set at the 75th percentile of the TPP distribution. For ROC analysis and for the comparison of the high and low TPP groups, TPP was determined by calculating the difference between TPV at the last follow-up visit and TPV at the baseline assessment. Likewise, the change in sP-selectin was defined as the difference between plasma P-selectin levels at the last follow-up and at baseline. Missing values were computed according to the last observation carried over principle. The predictive value of sP-selectin or hsCRP over time in terms of plaque progression was investigated by performing a receiver operating characteristic (ROC) analysis and determining the area under the curve (AUC) compared to the ESC-SCORE. To take the serial measurements of the biomarkers into account, the change in P-selectin was considered a predictor variable. Belonging to the high-TPP group was used as a classification variable. The effect of a combination of ESC-SCORE and sP-selectin progression on the prediction of plaque progression was evaluated by calculating a multiple logistic regression model with ESC-SCORE and changes in sP-selectin as newly added predictor variables. The fit of the model was assessed using the Hosmer–Lemeshow test. The calculated β-coefficients of the predictor variables were included according to the formula:

$$\ln\left(\frac{\hat{p}}{1-\hat{p}}\right)=\beta_{0}+\beta_{1}x_{1}+\beta_{2}x_{2}+\ldots +\beta_{k}x_{k}$$

The predictive performance was assessed by drawing a ROC-curve of the new prediction model and comparing the AUCs. We compared the AUCs by using the non-parametric DeJong test. After determining the optimal threshold for sP-selectin progression, we divided the study cohort into two groups: those with increasing sP-selectin levels and those with stable or decreasing sP-selectin levels. These two groups were then compared in terms of mean plaque progression over the course of the follow-up visits. In all statistical assessments, a two-tailed *p*-value of less than 0.05 was considered indicative of statistical significance. Statistical analyses were performed using SPSS Statistics Version 27.0 (IBM Corp, Armonk, NY, USA).

### 2.4. Ethical Issues

This study is an observational, non-interventional, single-center cohort study. The Ethics Committee of the University of Innsbruck (Project identification code—UN5048) approved the study protocol, and this study was conducted in accordance with the declaration of Helsinki. Each patient gave written and informed consent. Patient data were recorded and stored according to the General Data Protection Regulation of the European Union.

## 3. Results

Details of the baseline characteristics are shown in Table 1. The high-TPP group, with a mean age of 66 years, was significantly older. The majority of the study participants were male; however, in the low-TPP group, significantly more women were included.

In the high-TPP group, significantly more patients were hypertensive and received antihypertensive therapy. As expected, the high-TPP group showed significantly higher expected 10-year cardiovascular mortality as measured via the ESC-SCORE (4.0 in high-TPP vs. 2.0 in low-TPP, *p* < 0.001). All other parameters, including baseline sP-selectin and hsCRP levels, did not show statistically significant differences between the high and low TPP groups. The baseline parameters for plaque volume, IMT, ABI, and PWV are shown in Table 2.

Patients with high TPP had significantly higher total plaque volume (TPV), carotid plaque volume, and femoral plaque volume at baseline. Likewise, carotid IMT and PWV were significantly higher in the high-TPP group. In contrast, no difference was found for the ABI between the high and low TTP groups. With a median ESC-SCORE of around 3, the majority of the study participants belonged to the low- and intermediate-risk categories according to the ESC. After a mean follow-up of 3.4 years, the high-TPP group showed a significantly higher sP-selectin increase than the low-TPP group (Δ8.78 µg/mL in high-TPP vs. Δ−0.93 µg/mL in low-TPP, *p* < 0.001). The median increase in sP-selectin over all study visits was 4.98 µg/mL. Of our study population, 6 patients suffered from atherosclerotic disease in all three vascular beds, whereas 27 patients had two vascular beds and 96 patients had one vascular bed involved. Patients with a higher number of affected vascular beds had higher sP-selectin levels at baseline (no CVD: 42.4 µg/mL, one vascular bed: 45.2 µg/mL, two vascular beds: 49.1 µg/mL, three vascular beds: 52.6 µg/mL; *p* < 0.001). However, there was no statistically significant interaction between the number of CVDs and P-selectin levels over time, indicating that the initial significant difference between the groups remained stable. Each standard deviation increase in sP-selectin was significantly (*p* < 0.001) associated with a 46.09 mm^3^ higher total plaque volume. This effect remained on a significant level after adjustment for personal data (sex, age), classical cardiovascular risk factors (hypertension, dyslipidemia, diabetes, positive family history), and laboratory parameters (total cholesterol, HDL-cholesterol, LDL-cholesterol, and creatinine; see Table 3).

**Figure 2:**
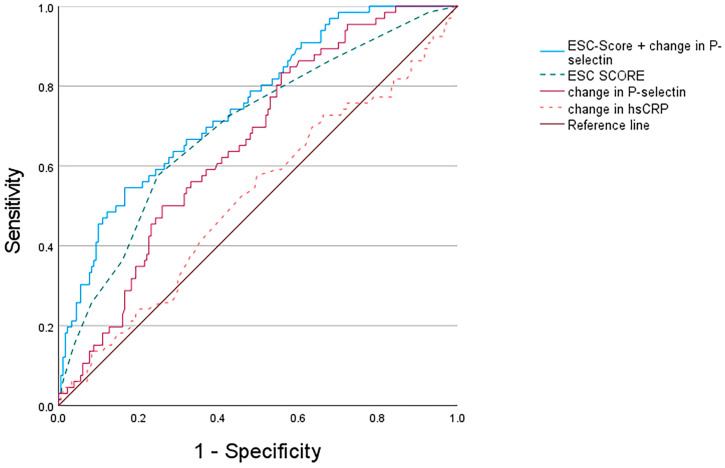
Receiver operating characteristics (ROC) curve for prediction of high plaque progression (high-TPP).

Conversely, the growth curve models of hsCRP did not show a significant association between changes in hsCRP and TPP (*p* > 0.05). Likewise, models that used the ABI as a dependent variable did not show a significant association with changes in sP-selectin (*p* > 0.05). In addition, we calculated the growth curve models using carotid plaque volume and femoral plaque volume as dependent variables. In both cases, the established model was statistically significant (*p* < 0.05). In the case of carotid plaque volume, each standard deviation increase in sP-selectin was associated with an increase of 22.04 mm^3^ (CI 95%: 18.67–26.03, *p* < 0.001). In the case of the femoral plaque volume, each standard deviation increase in sP-selectin was associated with an increase of 31.86 mm^3^ (CI 95% 26.97–37.62, *p* < 0.001). To establish the most suitable threshold for changes in P-selectin, we conducted a ROC analysis, yielding an area under the curve (AUC) of 0.65 (95% CI 0.58–0.72, *p* < 0.001). The optimal cut-off value, approximately Δ−0.0 µg/mL, was associated with a sensitivity of 74.7% and a specificity of 52.0%. With an AUC of 0.51 (95% CI 0.43–0.59; *p* > 0.05), hsCRP progression showed no significant predictive value. The ESC-SCORE had an AUC of 0.69 (95% CI 0.62–0.77, *p* < 0.001). Details are shown in Figure 2.

**Figure 3:**
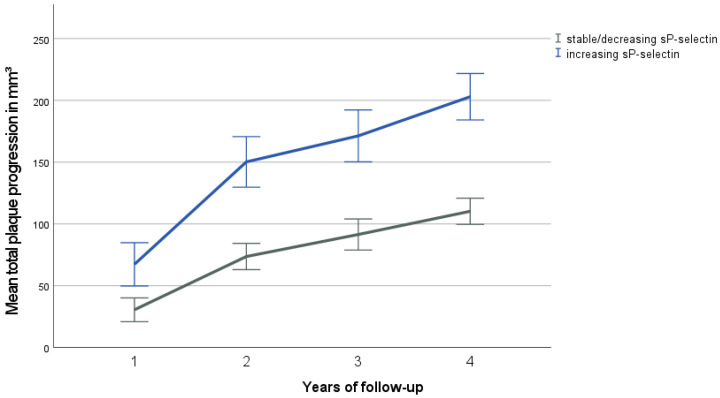
Mean total plaque progression by follow-up visit and sP-selectin-group (increasing vs. stable or decreasing sP-selectin levels). Follow-up visits occurred annually. Error bars represent a range of ±1 standard error of the mean (SEM). The group with increasing levels shows significantly higher plaque progression over the course of time (*p* < 0.05).

The multiple logistic regression model of the combined β-coefficients of ESC-SCORE and sP-selectin progression showed the highest predictive performance with an AUC of 0.75 (95% CI 0.68–0.81, *p* < 0.001). In the non-parametric DeLong test, the combination ESC-SCORE and sP-selectin change showed a significantly higher AUC than the ESC-SCORE alone (*p* = 0.005). For further statistical analysis, patients were divided into a group with increasing sP-selectin levels and a group with stable or decreasing sP-selectin values (Figure 3). The patient group with increasing sP-selectin levels showed a significantly higher mean plaque progression (202 mm^3^) over all study visits compared to patients with decreasing or stable sP-selectin levels (110 mm^3^, *p* < 0.001).

## 4. Discussion

In this study, we found that patients with increasing sP-selectin levels over a mean follow-up of 3.4 years showed a significantly higher TPP compared to patients with stable or decreasing sP-selectin levels. Our study population comprised patients with at least one CVRF or established CVD, most of them belonging to the low or intermediate risk category according to the ESC-SCORE [9]. In a study on 733 patients conducted by Tscharre et al., it was shown that elevated levels of sP-selectin were connected with an elevated risk of major adverse cardiac events (MACEs) in the long term after undergoing percutaneous coronary intervention (PCI) [32]. These results were recently supported by the research of Berg et al. in the DAPT trial, which showed that high sP-selectin levels had an increased risk of MACE after coronary intervention [33]. Aside from its role in acute coronary syndromes, Shen et al. found that sP-selectin levels were significantly higher in patients with established CAD compared to healthy people [34]. The role of P-selectin in the development of atherosclerosis is supported by the research of Johnson-Tidey et al., who observed that P-selectin is preferentially expressed in the endothelium overlying atherosclerotic plaques [17,18]. In addition, it was shown that P-selectin-deficient mice develop reduced fatty streaks, the first visible lesions in the pathogenesis of atherosclerosis [15]. In a recent study by Wang et al., the inhibition of P-selectin led to the stabilization of atherosclerotic plaques [19]. The use of a monoclonal antibody targeting P-selectin was shown to reduce myocardial damage in percutaneous coronary intervention following non-ST-segment elevation myocardial infarction [16].

These reported pro-atherosclerotic effects are consistent with the results of our growth curve models, where we could show that increasing sP-selectin levels are associated with increased atherosclerotic plaque volume. In the MESA study, Wassel et al. used the change in ABI as a surrogate parameter for atherosclerotic progression [35]. Using linear growth curve modeling, they found that increased baseline sP-selectin levels were associated with lower ABI values and a higher incidence of peripheral artery disease. However, unlike our study, these authors did not use serial measurements of sP-selectin. Furthermore, it was shown by Sillesen et al. that 3D plaque volumetry correlated stronger with coronary artery calcium score than the ABI, suggesting that plaque volume may be a better surrogate parameter for subclinical atherosclerosis than the ABI [26].

In addition to its pathophysiological role in the development of atherosclerosis, P-selectin has also been evaluated in terms of its prognostic value to improve risk stratification. In a recently published case-control study, the strongest association with MACE was observed for a composite parameter of sP-selectin, platelet count, and neutrophil extracellular trap markers [36]. Previously, we have shown that baseline levels of the acute phase protein neutrophil gelatinase-associated lipocalin (NGAL), but not hsCRP, correlate directly with TPV as measured by 3D ultrasound [27]. Baseline levels of fetuin-A, a serum protein involved in calcium homeostasis and inflammation, but not hsCRP, correlated inversely with TPP [28]. Using a pre-specified endpoint, we show that measurements of sP-selectin levels over time predict the progression of peripheral atherosclerosis and improve the predictive value of the ESC-SCORE in a multiple logistic model. Moreover, the well-studied biomarker hsCRP, although predictive in many other studies [33,37], was not strongly correlated to any of the parameters studied. In contrast to our results, Lee et al. reported serial measurements of hsCRP as a predictor of major cardiac and cerebrovascular events in patients after myocardial infarction [38]. We do not have an explanation for these discrepancies, but different biomarkers may reflect different steps in the pathogenesis and progression of atherosclerosis, depending on the characteristics of the patients studied. We conclude that serial measurements of sP-selectin appear to be predictive for atherosclerotic plaque progression in the peripheral arteries. In our study, changes in sP-selectin showed a strong association with TPP, carotid plaque progression, and femoral plaque progression. Similar to the results of the PESA study, our data highlight the role of the femoral territory in cardiovascular risk stratification [39].

Together with other studies, these observations suggest that 3D plaque volumetry may be a more precise surrogate parameter for subclinical atherosclerosis than IMT [25]. Since 3D ultrasound is not yet widely available, serial measurement of sP-selectin has the potential to become a useful biomarker for the progression of atherosclerotic disease.

The main strength of our study is the fact that it was specifically designed to test an association between sP-selectin and the progression of atherosclerosis, as assessed by measurements of plaque volume and IMT in the carotid and femoral arteries. In addition, this study has a prospective design with a relatively long follow-up (mean 3.4 years) for each patient. The major limitation is the relatively small sample size, although similar to comparable trials, and the conduction of the trial in only one center. Since most of our patients belonged to the low- or intermediate-risk category of the ESC-SCORE, it may not be possible to extrapolate the results to patients at higher risk.

## 5. Conclusions

In conclusion, we found that patients with increasing sP-selectin levels showed significantly higher plaque progression compared to patients with decreasing or stable sP-selectin levels. Serial measurements of sP-selectin levels appear to be useful in the cardiovascular risk assessment of patients with CVRF or confirmed CVD. Plasma biomarkers like sP-selectin that can be easily measured in blood samples have the potential to further improve risk stratification through prediction scores like the ESC-SCORE.

## Figures and Tables

**Table 1:** Study population baseline data. Data are presented with median values (interquartile range, IQR) or mean values (±standard deviation) for continuous variables and for categorical variables with absolute numbers and corresponding percentages.

	Study Population (*n* = 338)	Low-TPP (*n* = 256)	High-TPP (*n* = 82)	*p*-Value
Age, years	63.1 (±10.1)	62.0 (±10.4)	66.6 (±8.1)	<0.001
Female, n (%)	144 (42.6)	122 (47.7)	22 (26.8)	<0.001
Body mass index, kg/m^2^	25.3 (4.7)	25.2 (4.4)	26.2 (4.3)	n.s.
Hypertension, n (%)	218 (64.5)	154 (60.2)	64 (78.0)	0.003
Family history for CVD, n (%)	78 (23.1)	61 (23.8)	17 (20.7)	n.s.
Smoking, pack years	12.3 (±19.6)	11.4 (±19.06)	15.2 (±21.8)	n.s.
Hyperlipidemia, n (%)	295 (87.3)	224 (87.5)	71 (86.6)	n.s.
Diabetes mellitus, n (%)	47 (13.9)	36 (14.1)	11 (13.4)	n.s.
hsCRP, mg/dL	0.17 (0.29)	0.17 (0.32)	0.17 (0.27)	n.s.
sP-selectin, µg/mL	40.0 (24)	41.0 (25.1)	36.3 (20)	n.s.
Total cholesterol, mg/dL	196.5 (±46.4)	198 (±48.8)	189 (±37.5)	n.s.
LDL-cholesterol, mg/dL	115.0 (55.75)	119 (59.0)	106 (46.0)	n.s.
HDL-cholesterol, mg/dL	58.0 (26.75)	58.0 (28.0)	58.0 (26.0)	n.s.
Triglyceride mg/dL	128.0 (85.0)	132 (88)	118 (76)	n.s.
eGFR, mL/min/1.73 m^2^	76.6 (±15.5)	77.59 (±15.9)	73.53 (±13.9)	n.s.
Antiplatelet therapy, n (%)	157 (46.4)	118 (46.1)	39 (47.6)	n.s.
Lipid lowering therapy	192 (56.8)	144 (56.3)	48 (58.5)	n.s.
Antihypertensive therapy	195 (57.7)	139 (54.3)	56 (68.3)	0.02
ESC SCORE	3.0 (3.0)	2.0 (3.0)	4.0 (2.5)	<0.001
CAD, n (%)	106 (31.4)	82 (32.0)	24 (29.3)	n.s.
CRVD, n (%)	34 (10.1)	25 (9.8)	9 (11.0)	n.s.
PAD, n (%)	28 (8.3)	20 (7.8)	8 (9.8)	n.s.

TPP—total plaque progression; CVD—cardiovascular disease; CAD—coronary artery disease; hsCRP—high-sensitivity C-reactive protein; LDL—low-density lipoprotein; sP-selectin—soluble P-selectin; HDL—high-density lipoprotein; eGFR—estimated glomerular filtration rate, ESC—European society of cardiology, CRVD—cerebrovascular disease, PAD—peripheral arterial disease, n.s.—not significant.

**Table 2:** Distribution of baseline values for carotid intima-media thickness, total plaque volume, ankle brachial index, and pulse wave velocity is depicted using medians and their corresponding interquartile ranges (IQR).

	Study Population (n = 338)	Low-TPP (n = 256)	High-TPP (n = 82)	*p*-Value
TPV, mm^3^	296.5 (462.25)	248 (446.5)	457.0 (443.5)	<0.001
Carotid plaque volume, mm^3^	86.0 (230.25)	72.0 (199.75)	125 (235)	0.006
Femoral plaque volume, mm^3^	174.0 (282.0)	135.5 (262)	218.5 (278)	<0.001
Carotid IMT, mm	0.72 (0.19)	0.72 (0.19)	0.76 (0.23)	0.003
ABI	0.91 (0.15)	0.91 (0.16)	0.91 (0.15)	n.s.
PWV, m/s	5.9 (2.4)	5.72 (2.18)	6.4 (2.5)	0.004

ABI—ankle brachial index, PWV—pulse wave velocity, TPP—total plaque progression, TPV—total plaque volume, IMT—intima-media-thickness, n.s.—not significant.

**Table 3:** Association of sP-selectin and plaque progression.

	Plaque Progression in mm^3^ (95% CI)	*p*
sP-selectin	46.09 (24.75–67.43)	<0.001
+personal data	38.64 (19.12–58.17)	<0.001
+CVRF	30.19 (12.27–48.11)	<0.001
+laboratory parameters	24.15 (7.64–44.48)	0.005

Plaque progression per standard deviation P-selectin (standard deviation sP-selectin 20.13 µg/mL) after stagewise inclusion of additional covariates. Personal data = sex, age; CVRF = smoking status, hypertension, diabetes, hyperlipidemia, family history; laboratory parameters = total cholesterol, HDL-cholesterol, LDL-cholesterol, creatinine.

## Data Availability

This published article contains all the data generated or analyzed during the study within the manuscript.
